# Monitoring the Depth of Anaesthesia

**DOI:** 10.3390/s101210896

**Published:** 2010-12-03

**Authors:** Bojan Musizza, Samo Ribaric

**Affiliations:** 1 Department of Systems and Control, Jožef Stefan Institute/Jamova 39, SI-1000 Ljubljana, Slovenia; E-Mail: bojan.musizza@ijs.si; 2 Institute of Pathophysiology, Faculty of Medicine, University of Ljubljana/Zaloška 4, SI-1000 Ljubljana, Slovenia

**Keywords:** consciousness, cognitive binding, general anaesthesia monitors, soft sensors, general anaesthesia

## Abstract

One of the current challenges in medicine is monitoring the patients’ depth of general anaesthesia (DGA). Accurate assessment of the depth of anaesthesia contributes to tailoring drug administration to the individual patient, thus preventing awareness or excessive anaesthetic depth and improving patients’ outcomes. In the past decade, there has been a significant increase in the number of studies on the development, comparison and validation of commercial devices that estimate the DGA by analyzing electrical activity of the brain (*i.e.*, evoked potentials or brain waves). In this paper we review the most frequently used sensors and mathematical methods for monitoring the DGA, their validation in clinical practice and discuss the central question of whether these approaches can, compared to other conventional methods, reduce the risk of patient awareness during surgical procedures.

## 1. Introduction

### 1.1. Why is it necessary to monitor the depth of general anaesthesia?

Many surgical procedures would not be possible without the patient entering a state of general anaesthesia (GA). The essential features of a successful GA, displayed by the patient, are a reversible loss of consciousness with a lack of movement, a lack of awareness, unresponsiveness to painful stimuli and a lack of recall of the surgical intervention. Inadequate GA may lead to intraoperative awareness with recall (due to patient underdosage) or to prolonged recovery and an increased risk of postoperative complications for the patient (due to overdosage). An important contributing factor to inadequate GA is our current limited ability to evaluate the levels of consciousness [1].

The incidence of awareness has been reduced from about 1–2% in unselected patients in the 1980s [2] to about 0.1% at present [3–6]. However, certain surgical procedures (for example caesarean section or cardiac surgery) or high risk patients have a substantially increased risk of awareness [7]. The consequences of intraoperative awareness range from an absence of prolonged after-effects to a post-traumatic stress disorder [7]. In comparison, worldwide anaesthesia-related mortality rates during the 1990 to 2006 period ranged between 75 (in Thailand) and 1 (in the USA or Japan) per 100,000 anaesthetic procedures. The main causes of anaesthesia-related mortality were problems with airway management and cardiovascular events related to anaesthesia and drug administration [8].

**Figure 1.**
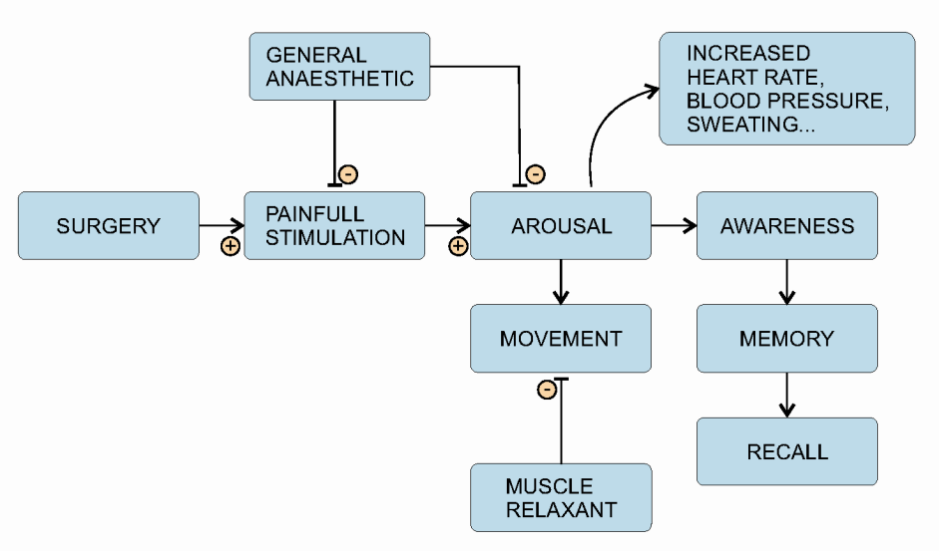
The relationship between surgical stimuli, general anaesthetics and awareness.

Important signs of inadequate patient GA (Figure 1), signs which develop in response to stress or painful stimuli, are movement, increased breathing or heart rate and increased blood pressure [9]. These warning signs are often attenuated since many surgical procedures require administration of a muscle relaxant or the patient receives medication that affects the heart rate and blood pressure [9]. Avoidance of muscle relaxants is no guarantee of avoiding awareness. Studies have reported patients with awareness under non-relaxant anaesthesia [3–5]. Fortunately, such awareness is very rare and very rarely reported as unpleasant [3].

It is generally believed that awareness will not occur if GA is maintained with a volatile agent delivered to the patient’s blood at a concentration of at least 0.5 minimum alveolar concentration (MAC) [9]. However, for some patients, the use of total intravenous anaesthesia techniques is more appropriate [3,10–13]. Individual variations in patient drug requirements will also lead to occasional over dosage or under dosage with both volatile-based and intravenous-based techniques [9,11]. Failure to adjust the anaesthetic requirements to individual variations in patient drug requirements and to the intensity of pain stimulation during a specific surgery procedure will also lead to over dosage or under dosage with both volatile-based and intravenous-based techniques [9,11].

### 1.2. Monitoring anaesthetic delivery is not the same as monitoring anaesthetic effect

The potencies of general anaesthetics range over at least five orders of magnitude [14]. However, for any given anaesthetic, the concentration at which consciousness is lost is well defined. For intravenous or volatile anaesthetics, the transition from the conscious to the unconscious state occurs abruptly over a change in concentration of only a factor of less than 1.6 [14]. The physiological mechanism for this abrupt switch between consciousness and unconsciousness was explained by the intrinsic bi-stability of thalamocortical neurons coupled with the reciprocal inhibitory connections between hypothalamic sleep-promoting centres and the arousal nuclei in the midbrain and the brainstem, which would tend to favour rapid transitions between high and low states of ascending arousal [14].

Administration of at least 0.5 MAC of a volatile anaesthetic agent should prevent awareness [15] and most anaesthetists employ this approach to prevent awareness in their daily practice [9]. Similar results can be obtained with total intravenous infusion devices that incorporate predicted plasma and effect site concentrations [16]. However, since we measure the volatile anaesthetic’s concentration in the lungs and only simulate the concentration of intravenous anaesthetic drugs, we do not know exactly and accurately what anaesthetic concentration is reaching the brain at all times throughout the surgical procedure. In other words, volatile-based and intravenous-based anaesthetic devices measure or predict drug concentration not drug effect [9].

### 1.3. Monitoring anaesthetic depth

The transition from a state of wakefulness to a state of GA is accompanied by profound changes in the brain’s spontaneous electrical activity recorded from electrodes placed on the scalp (an electroencephalograph or EEG). The EEG reflects the compound synaptic activity of excitatory and inhibitory post-synaptic potentials generated by cortical neurons [17]. However, to achieve an optimal level of GA, it is not possible, nor practical, to adjust the delivery of volatile or intravenous anaesthetics on the basis of an on-line EEG [17]. Only the advances in computer hardware and signal processing algorithms have enabled the processing of EEG signals (Table 1) and the development of monitors that estimate the depth of general anaesthesia (DGA) on a near to on-line basis [9,17–20]. To understand how DGA monitors work, their respective advantages and disadvantages, it is necessary to understand the theoretical foundations that contributed to their design and this also includes a basic understanding of the relation between GA and consciousness.

## 2. Consciousness and General Anaesthesia

Consciousness can be defined as explicit awareness. Awareness implies that the brain is aroused and that a person has specific perceptual qualities of an experience (e.g., a hot chocolate drink). The term “explicit” distinguishes conscious awareness from cognitive processes in the brain that are implicit or unconscious. Explicit awareness does not necessarily imply that the patient will also have explicit recall, for example the recall of a surgical intervention [21]. The key anatomic structures of the central nervous system (CNS) that contribute to the state of consciousness are: the brain stem, the pons, the thalamus (thalamic nuclei) and the brain cortex with their connecting neural pathways [21,22].

### 2.1. Molecular and cellular actions of general anaesthetics

There are two types of general anaesthetics: (a) intravenous agents (e.g., propofol), generally administered together with sedatives or narcotics and (b) volatile agents (e.g., sevoflurane). Both types of anaesthetics modulate the permeability of ion channels that regulate synaptic transmission and membrane potentials in key regions of the CNS [14,23,24]. All general anaesthetic agents are relatively apolar, to be able to cross the blood-brain barrier, and interact with their target (*i.e.*, receptor) through weak polarization forces and hydrogen bonding [25]. The binding of a general anaesthetic to its receptor leads to neuron hyperpolarisation due to increased inhibition or to decreased excitation thus altering neuronal activity [26]. The actions of a general anaesthetic on the molecular level are reflected on the brain’s electrical activity as a transition from the low voltage, high-frequency pattern of wakefulness (known as activated EEG), to the slow-wave EEG of the deep, non-rapid eye movement (NREM) sleep, and finally to an EEG burst-suppression pattern [27].

General anaesthetics act on different targets, *i.e.*, receptors, for example γ-aminobutyric acid (GABA) type A, nicotinic acetylcholine, and glutamate receptors (e.g., *N*-methyl-d-aspartate receptors) in the brain, as well as glycine receptors in the spinal cord [28,29]. A clear distinction has to be made between the actions of general anaesthetics and the mechanism of general anaesthesia. The actions of general anaesthetics on the brain receptors are well known; the process by which these actions translate into general anaesthesia (*i.e.*, the mechanism of general anaesthesia) is not well understood [21].

### 2.2. Linking the cellular and molecular actions of general anaesthetics with the mechanism of general anesthesia

**Figure 2.**
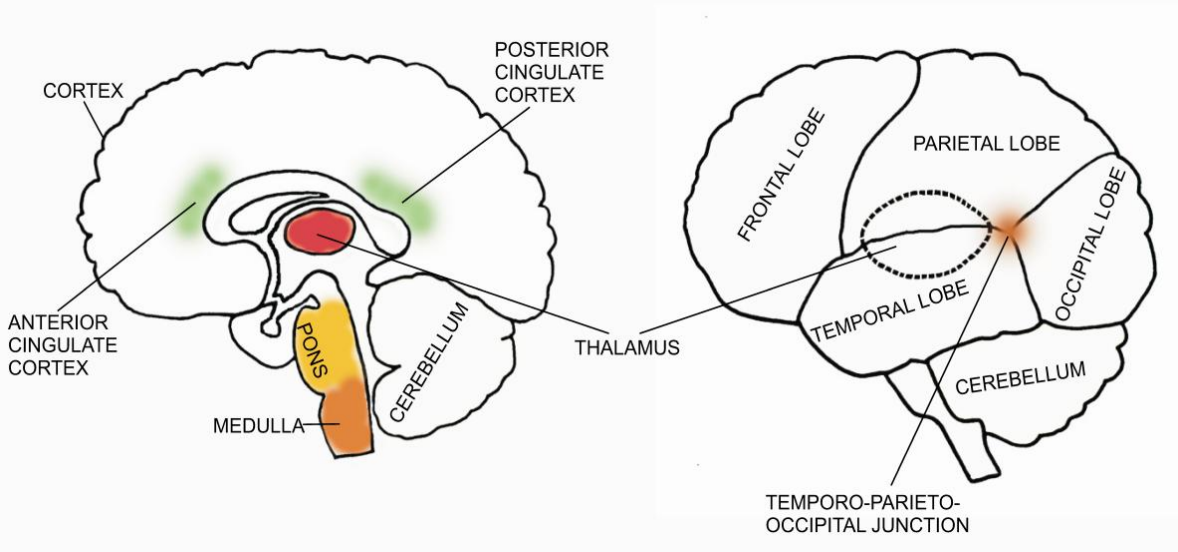
Key regions (shaded in colour) in the central nervous system that contribute to the state of consciousness.

Distinct anaesthetic properties are correlated with distinct sites in the CNS; for example, loss of consciousness is associated with the cerebral cortex, loss of memory with the limbic system and immobility and analgesia with spinal cord effects [21]. General anaesthetics modulate the activity of several CNS structures including the spinal cord, brain stem, pons, thalamic nuclei and specific regions of the brain cortex (Figure 2). The effects of general anaesthetics on the brain, which lead to loss of consciousness, are best understood at the level of the cortex and the thalamus.

The thalamus has numerous connections to the cortex (*i.e.*, the thalamo–cortical pathway, TCP) and individual thalamic nuclei modulate the activities of specific cortical regions. In addition, the cortex sends connections back to the thalamus (*i.e.*, the corticofugal pathway, CFP) which greatly outnumber the thalamo–cortical connections [14]. The corticofugal connections provide a continuous depolarization of the thalamo–cortical neurons, during the brain’s wakeful state, preventing them from entering a synchronized, oscillatory state. The activity of the TCP is in balance with the activity of the CFP through a positive feedback loop [14]. A reduced activity of the CFP promotes the activity of the TCP, the TCP neurons enter into an oscillatory mode which further reduces the activity of the CFP and the subject enters into a sleep-like state (*i.e.*, hypnosis). Most anaesthetics (ketamine is an important exception) produce EEG patterns that are reminiscent of spindle and δ waves, the EEG landmarks of falling asleep and deep, dreamless NREM sleep, respectively. Most anaesthetics initially produce high-frequency oscillations followed by a lower frequency and a higher amplitude EEG pattern at or beyond the point at which consciousness is lost [14]. It has been suggested that loss of consciousness occurs when information transfer through the thalamus is disrupted during GA following a reduction of thalamic metabolism and blood flow [14,30,31]. It has also been suggested that the thalamus could behave as a consciousness switch [30]. However, thalamic activity does not decrease with all anaesthetics; some general anaesthetics (e.g., ketamine) increase thalamic metabolism and some general anaesthetics (e.g., sevoflurane) substantially reduce thalamic activity without loss of consciousness when applied in low doses and lead to unconsciousness at high doses [32,33]. All anaesthetics will produce sedation at moderate doses. Therefore, it is more likely that the effects of general anaesthetics on the thalamus reflect global cortical activity rather than the thalamus acting as a consciousness switch [1,22]. It has been suggested that efficient communication among cortical areas (especialy among the long range cortico–cortical connections) requires a functional thalamic connection [34,35]. A functional thalamic disconnection of the brain cortex, during GA, could still lead to cortical arousal, but not to consciousness [22].

All cortical areas are not equally important for the induction of unconsciousness by general anaesthetics. A complex comprising the lateral temporo–parieto–occipital junction and the mesial cortical core is most likely the final common target for anaesthetic-induced unconsciousness [22]. Anaesthetics disrupt two essential cortical functions: cortical integration and cortical information capacity. Anaesthetics may disrupt cortical integration by acting on structures that facilitate long-range cortico-cortical interactions [22]. Anaesthetics disrupt synchronization of brain areas by delaying feedback interactions among distant (not adjacent) brain areas within the cortex [36] or between small-world networks with predominant local connectivity and a few long-range connections—for example the cortico–thalamic system [37]. The cortico–thalamic system produces global responses during deep anaesthetic unconsciousness. However, these responses are limited to a stereotypic burst-suppression pattern, with a corresponding loss of information, thus creating a system having only two possible states (*i.e*., on or off) [22,38,39].

General anaesthetics also inhibit the excitatory arousal pathways originating in the brain stem and pons or potentiate the sleep pathways that control them [14]. The brain stem and pontine nuclei have been known to be essential in maintaining cortical arousal and form the so called ascending reticular formation [14].

### 2.3. Unity of consciousness, cognitive binding and the mechanism of GA

The concept of unity of consciousness was introduced by the 18th century German philosopher Immanuel Kant [21,40]. According to Kant, for a perception to have a meaning its distinct qualities must be brought together into a single experience. Kant postulated the existence of a mechanism that synthesized the diverse and complex processes of the mind to generate a single perception *i.e.*, the unity of consciousness [21]. The visual system is the best understood model of how the brain processes and integrates sensory information. The key question, also known as the cognitive binding problem [41] that arose from these studies was: “How does the brain synthesize the elements of visual processing, located at diverse locations of the cortex, to generate a unified visual perception?”.

Cognitive binding is necessary, but not sufficient, for the brain to sustain the unity of consciousness. The proposed mechanisms for cognitive binding in the brain [42,43] are binding by convergence (transmission of information from primary processing areas to another region of the brain for integration), binding by assembly (binding information in a group of inter-related neurons whose connections grow stronger when repeatedly firing together) and binding by synchrony (coordination of neural firing in time associated with neural events at the frequency of about 40 Hz). The evidence from GA and sleep studies suggests that loss of consciousness is associated with a breakdown of cortical connectivity, a diminished cognitive binding, a reduction of the repertoire of cortical activity patterns (*i.e.*, the predominance of stereotypic responses) and a loss of information [1,21].

Recently, a “cognitive unbinding” paradigm of GA was proposed to explain the actions of general anaesthetics [44–46]. To summarize, consciousness is not an all-or-none property, but is graded; it increases in proportion to a system’s repertoire of distinguishable states. The shrinking of the field of consciousness during GA is proportional to the reduction of the brain’s repertoire of distinguishable states. A transition point is reached during GA when a nonlinear collapse of cognitive binding leads to loss of consciousness [1,21]. A practical application of the concept of cognitive unbinding to GA, the “anaesthetic cascade” theory, was proposed by John and Prichep [47]. The anaesthetic cascade theory attempts to link progressive suppression of consciousness during GA with the action of general anaesthetics on specific brain regions.

### 2.4. The anaesthetised patient

The initial signs of GA (stage 1 anaesthesia) can be a state similar to drunkenness, analgesia (the inability to feel pain while still conscious), amnesia (loss of memory), distorted time perception or increased sleepiness. With higher anaesthetic doses, a patient fails to move in response to a verbal command and is considered to be unconscious. However, unresponsiveness during GA can occur without unconsciousness. For example, paralyzing agents used to prevent unwanted movements during anaesthesia do not remove consciousness [48]. Most general anaesthetics will cause a global deactivation of the brain at higher doses [1]. The exceptions are dissociative anaesthetics, like ketamine, that increase global brain metabolism, especially in the thalamus [32]. The ketamine induced changes in global brain metabolism are reflected in the responses of the anaesthetised patient. Ketamine causes loss of motivation to follow commands at lower doses [49]; at higher doses ketamine causes a characteristic state in which the eyes are open and the face takes on a disconnected blank stare consistent with the deactivation of executive circuits in anterior cingulate cortex and basal ganglia [1]. At doses near the unconsciousness threshold, some anaesthetics block working memory and patients may fail to respond because they immediately forget what to do [50]. Also, some patients under general anaesthesia can carry on a conversation using hand signals, but postoperatively deny ever being awake during the surgical procedure [51]. Surgery is performed when the patient is perceived to reach stage 3 general anaesthesia. Increasing doses of modern general anaesthetics (*i.e*., an overdose) do not cause brain death [52] directly but lead to cessation of spontaneous ventilation and impairment of cardiovascular function (stage 4 anaesthesia). This stage is lethal without proper cardiovascular and respiratory support, which is standard in a modern operating theater. Therefore, monitoring the DGA is essential to tailor the optimal dose of general anaesthetic to the individual patient thus reducing the incidence of intraoperative awareness or preventing the effects of an overdose of general anaesthetic. Although there are several “brain-function monitors” available they have a limited ability to detect directly the presence or absence of consciousness, especially around the transition point when loss of consciousness occurs [1]. This issue will be discussed in detail in Section 4.

## 3. Key Considerations when Designing and Evaluating DGA Monitors

The search for a DGA monitor, that would enable objective, reproducible and continuous measurement of anaesthetic depth, has lead to the development of EEG (electroencephalogram) or AEP (acoustic evoked potentials) based monitors [9,17–19]. These enable continuous monitoring even during conditions during which a patient has lost all responses to external stimuli (to non-painful or painful). When interpreting the results of EEG or AEP based monitors it is important to distinguish the influences on EEG activity from the influences on EEG measurements. The EEG activity is influenced by GA alone, by general anaesthetics [53], by physiological states or by other drugs [54–60] (different drugs lead to different changes in EEG activity).

**Figure 3.**
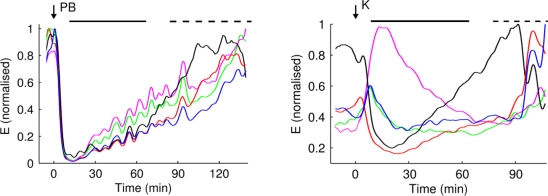
Relative amplitude changes in EEG frequency bands during pentobarbital (PB) and ketamine (K) anaesthesia in an animal model. Legend to colour coded EEG wave bands: black (δ), red (θ), blue (α), green (β) and violet (γ). The solid horizontal line denotes a deep general anaesthesia; the dotted horizontal line denotes a shallow general anaesthesia.

Most general anaesthetics (e.g., propofol, etomidate, pentobarbital or halothane) produce anaesthesia by increasing the activity of inhibitory γ-aminobutyric acid type A receptors (GABA_A_ receptors, a type of ligand gated ion channels) in the brain [1,62]. This is reflected in a general reduction of EEG activity, accentuated with progressive anaesthetic concentrations (Figure 3). Exceptions to this rule are ketamine, xenon and nitrous oxide that produce anaesthesia by interacting with other brain receptors, mainly, but not exclusively, by inhibiting excitatory *N*-methyl-d-aspartate (NMDA) brain receptors [1]. These three general anaesthetics produce different changes in EEG activity than the so called “GABA_A_-type anaesthetics” (e.g., halothane). The division of general anaesthetics between “GABA_A_-type anaesthetics” and “non GABA_A_-type anaesthetics” is only a convenient simplification since most, if not all general anaesthetics, modulate (*i.e.*, potentiate or inhibit) the activity of several cell membrane channels in the brain. For example, sevoflurane causes a major potentiation of the activity of GABA and glycine activated ion channels and a major attenuation of the activity of NMDA, 5-HT_3_ (5-hydroxytryptamine) and AMPA (α-amino-3-hydroxyl-5-methyl-4-isoxazole-propionate) receptor channels.

Contrary to expectations, ketamine *per se* does not change the BIS (bispectral analysis) index value even when patients are unconscious [61]. Administration of ketamine, during total intravenous anaesthesia with propofol and fentanyl, caused a paradoxical increase in the BIS value [62]. The explanation for this paradoxical increase in BIS value was that BIS may have detected a concomitant and causally unrelated cerebral hypoperfusion during the combined ketamine-propofol-fentanyl anaesthesia [63–65] since ketamine does not reduce the baseline BIS; also BIS in an empiric score, not a pure mathematical construct of raw EEG. Hypoperfusion of the brain can cause changes in EEG activity that are visible in the raw EEG record, or more subtle EEG changes that can be detected only after mathematical analysis. Supporting this explanation is the observation that during ketamine anaesthesia the BIS value changed (presumably due to cerebral ischemia) in a patient undergoing surgery of the aorta [65]. Ketamine has no effects on AEP [67–69], consistent with the observed effects of ketamine anaesthesia on BIS values. The effects of nitrous oxide on the raw EEG or on the BIS value are varied and therefore unpredictable [61,62,66,70,71]. Xenon produces BIS values similar to GABA_A_-type anaesthetics, but some patients with a BIS value suggesting deep anaesthesia, do awake from xenon anaesthesia [72]. Therefore, current EEG (e.g., BIS) or AEP based anaesthesia monitoring devices are not able to reliably assess the patient’s depth of anaesthesia when ketamine, xenon or nitrous oxide are used.

β-activation and burst suppression are EEG observed changes in the normal brain activity during GA. Most general anaesthetics elicit an increase in β EEG frequency band, between 13 and 30 Hz [18]. Therefore, EEG-based depth of anaesthesia monitors have to employ mathematical algorithms to compensate for this “β activation”, to prevent this pattern of EEG activation being reported by DGA monitors as arousal [18,73,74]. Burst suppression (BS) represents an EEG pattern frequently seen in healthy brain at deep levels of anaesthesia [18]. This pattern is composed of episodes of electrical quiescence (suppression) alternated with high frequency, high amplitude electrical activity (bursts). The duration of suppression periods increases with anaesthetic depth. Commercially available DGA monitors have mathematical algorithms that prevent the “burst” intervals to be interpreted as arousal—the patient being awake and reactive to stimuli [75–80].

Physiological conditions, such as age [81,82], race [83,84], gender [85–87], low core body temperature [88], acid-base imbalances [89–91], low blood glucose [92,93] or brain ischemia [62] also have a significant effect on the raw EEG. Drugs administered to the patient can influence the EEG either through a direct mechanism on the raw EEG [94,95] or by interacting with the absorption, distribution and elimination of the general anaesthetic drug [96]. Of particular consideration are neuromuscular blocking agents (used to prevent patient movement and to reduce muscle tone) that have a distinct effect on muscle electrical activity and indirectly on the signal quality of the EEG measurement [97,98]. Useful information can only be extracted from the EEG after pre-processing and analysis in time and frequency domain with numerous mathematical techniques (for details see Section 4).

The quality of EEG records is mainly influenced by internal or external sources of electromagnetic waves. This is the reason for the limited value of raw EEG records to monitor the DGA. Numerous sources of interference can distort EEG measurements. Well know sources of interference are the electrical activity of head muscles [99,100] or heart pacemaker [101]; other common sources of electromagnetic waves are hot air blanket systems [102] and electro coagulation needles [103,104]. The most important source of interference on EEG is the electrical activity of the muscles (measured with the EMG—the electromyogram). The fast electrical rhythms in the γ range (30–100 Hz) in scalp (but not intracranial) recordings are predominantly due to EMG activity in conscious humans [105]. In addition, electrical rhythms in the γ frequency range recorded from the scalp are inducible by mental activity and are largely due to EMG that is un-related to cognitive effort [105]. Since experimental evidence links the γ EEG frequency band with cognition and by extension to the level of consciousness (see Section 2.3 above) it would be possible to confuse changes in the patient’s EMG activity during GA with the patient’s state of consciousness during induction or recovery from GA (EMG activity alone has been used as a marker of anaesthetic depth, but difficulties in interpretation make this approach impractical).

Auditory evoked potentials (AEP) are the response of the auditory pathway to sound stimuli [17]. An AEP is calculated by repeatedly applying an auditory stimulus to the patient and averaging EEG periods that immediately follow each stimulus, thus eliminating the non-stimulus-related portion of the EEG and preserving the specific evoked potentials [17]. These potentials can be divided into several segments according to the anatomical area of its origin and the time elapsed since the stimulus. The middle latency auditory evoked potential, 40 and 60 ms after stimulation, represents neural activity within the thalamus and primary auditory cortex and is most often used as a measure of the anaesthetic effect [17,106–108]. Compared to the raw EEG, AEP are less sensitive to artefacts that are random in time of occurrence since these artefacts tend to be eliminated from the EEG signal by repeated averaging [17].

There is no agreement on the minimum level of performance for EEG or AEP DGA monitors although many validation studies have been performed (for details see reference [17]). A consensus on the validation methodology is urgently needed since we still have no direct measure of consciousness available in clinical practice and thus no gold standard against which to test EEG or AEP derived indices of aesthetic depth [9,19,20]. A consensus on the validation method would enable to define a minimal level of performance in clinical and experimental settings and facilitate the comparison of the vast number of reported trials [19].

During GA, different patients loose consciousness at different (lower or higher) drug concentrations. Also changes in the patient’s age or general health may require adjustment of anaesthetic drugs. This variability in anaesthetic drug concentration is caused by a variability in the physiological effects of drugs on the body (differences in pharmacodynamics) and by a variability in drug metabolism in the body (differences in pharmacokinetics). Therefore it is important to establish whether a DGA monitor detects relevant clinical changes (*i.e.*, the transition point between awareness and loss of consciousness) independently of the amount of drug needed for that effect to occur, since different patients will reach this transition point at different anaesthetic concentrations [9,19,20].

Improvement of patient outcome should be an essential feature of any DGA monitor. Short term benefits (e.g., the amount of anaesthetics used, the recovery time or the length of stay in the recovery room) are easy to quantify but of equal importance are the long term benefits like, reduced risks of awareness or mortality [19]. What will ultimately decide the fate of any DGA monitor is its cost-benefit ratio to the patient and to society which is validated only by large multicentre trials [19].

Therefore, a systematic approach to evaluating DGA monitors is necessary to deal with all of the above mentioned considerations. Heyse *et al*. proposed a five step approach to validating the ability of anaesthesia monitors to monitor the depth GA [19]. These five steps are: (a) validation of the index for detecting clinical signs of anaesthesia during anaesthesia induction and recovery, (b) pharmacokinetic-dynamic validation, (c) validation of performance under clinical conditions, (d) demonstrating improvement of outcome and (f) demonstrating cost-benefit effectiveness.

## 4. Basic Design Characteristics of Current DGA Monitors

Soft sensors are used to simultaneously process signals, that are observed over time and contain random variations and other inaccuracies, to produce values based on signal interactions. Examples of software algorithms that can be seen as soft sensors are, e.g., Kalman filters [109], neural networks [110] or fuzzy computing [111]. Implementing a soft sensor solution requires a sound understanding of the process as a whole and specifically of the variable measurements. The procedure for implementing a soft sensor solution includes: data and information collection, data analysis and modelling, soft sensor model design, new process knowledge and current expert knowledge amalgamation, implementation and commissioning and performance monitoring.

From the engineers’ perspective the monitoring of the DGA is essentially a monitoring and control system. The key element in this system is a “soft” sensor or virtual sensor consisting of a hardware sensor (*i.e.*, EEG or AEP electrodes) that are integrated with custom hardware and software to produce a dimensionless number (e.g., on the scale from 0 to 100) indicating the level of general anaesthesia. This number is then used by the anaesthesiologist as a reference point to decide whether the level of general anaesthesia is appropriate and if not, to increase or decrease the amount of general anaesthetic.

### 4.1. Monitoring the DGA

The process of monitoring depth of GA and administration of a general anaesthetic during surgery is a closed-loop control system where the human is responsible for reasoning and action. The anaesthetists play the roles of controller and actuator by deciding on the amount of anaesthetic and when to administer it. On the other hand, the activity of monitoring is performed automatically by commercially available DGA monitors. Together they form a closed-loop control system.

In the last decade several experiments have been performed, where anaesthesia was controlled in a closed-loop control system without human interference [114–117]. Although these experiments were relatively successful and represent a proof of principle, there is still a long way to the autonomous commercially available depth of GA control system. Additionally, these experiments raise new questions, which have been only partially addressed so far. For example, the challenge is now to establish fully the safety, efficacy, reliability and utility of closed-loop anaesthesia for its adoption into the clinical environment [119]. Moreover, these systems still have to go trough rigorous testing and validation. Additionally, while the closed-loop system can maintain a constant target sedation level it cannot adjust the level of anaesthetic delivery in advance to meet the future needs during a surgical procedure. On the other hand, this scenario can easily be predicted and successfully resolved by the anaesthesiologist in communication with the surgeon. Therefore, a closed-loop control system for automatic administration of anaesthetic is feasible, but some important practical issues have to be addressed.

The proposed control systems are most often built around a well established BIS monitor, which is now standard equipment for GA monitoring. However, in the last decade the BIS monitor has undergone rigorous testing, which has shown several drawbacks [118,119]. Therefore the problem of constructing an ideal DGA is still unsolved.

It is a matter of debate how consciousness is formed let alone how it should be measured. In other words, we currently rely on indirect measurable quantities, which give us a relatively reliable measure of the state of awareness. Several algorithms and methods have been developed for assessment of the DGA in the last decade. Their common denominator is the reduction of information, which comes from one or more complex signals, into a single feature, which reflects the state of consciousness. This concept puts DGA monitoring systems in the domain of software sensors [109,110].

### 4.2. EEG processing

#### 4.2.1. Time domain analysis

The time domain analysis deals with the changes of the EEG signal in time. The time domain analysis can be based on some morphological features of the signal, e.g., burst suppression. On the other hand the analysis can be purely statistical, where mean and/or variance can be studied. Furthermore, EEG is a random non-stationary signal, so the information theory approach can be applied, where information theory functionals like entropy can be used to determine the degree of randomness of the EEG signal.

##### Burst suppression

During deep anaesthesia the EEG may develop a characteristic pattern, which is known as burst suppression (BS). It is characterised by alternating periods of normal to high voltage activity changing to low voltage. This kind of activity is usually seen when the concentration of anaesthetic drug is high [120,121]. To quantify this effect the burst suppression ratio (BSR) is calculated. BSR is calculated as a ratio between the duration of suppression and the duration of bursting.

##### Statistical modelling with autoregressive models

In this case we can approach the problem with techniques that construct models entirely from the measurements or time series, which come from the system [122,123]. Since our knowledge about the system is limited, we only try to infer the basic statistical properties of the system. This can be achieved by using linear stochastic models like autoregressive (AR) models. AR models are based entirely on time series data; therefore the terms they contain do not have a meaningful physical interpretation. However, a successful model can reproduce the statistical properties of original data.

##### Information theory and dynamical systems

Many approaches exist for quantifying different aspects of stochastic signals [124–127]. One such approach is by using entropy to quantify the regularity of the system. The entropy can be applied to time domain signals as well as to the frequency domain power spectrum. In the latter case it is referred as the spectral entropy. Entropy describes the irregularity, complexity of the signal, e.g., a signal which is periodically alternating between two fixed amplitudes is completely predictable and has entropy value of zero. On the other hand, a signal which is generated by some random process has greater complexity and higher entropy. Also, the entropy has the property that it is independent of an absolute scale, e.g., amplitude and frequency, additionally; the entropy can detect non-linear features of a signal, which makes it an appropriate method for studying EEG signals.

##### Symbol Analysis

Complex systems and their time series can also be studied with a set of methods, which rely on symbolic representation of the data. This approach is a crude reduction of information; nevertheless, it can be used to enhance the relevant features of the data. It is particularly useful to track qualitative changes in the dynamics of the time series [122,123]. When applied to the complex signals like EEG the symbol dynamics approach can characterise the non-linear features of the EEG, which can then be linked to various depth of sedation.

#### 4.2.2. Frequency domain analysis

The frequency domain analysis is an important approach to signal analysis, where the signal features are examined in respect to frequency. With Fourier analysis one obtains amplitude and a phase spectrum, which is analogous to the histogram of amplitudes and phase angles of the signal components [128].

##### Fourier transform

The Fourier Transform (FT) plays a central role in the implementation of a variety of digital signal-processing algorithms, e.g., filtering and spectral analysis [128]. When applied to the analysis of the EEG it is mostly used to calculate and derive different spectral features, which are important to the depth of GA: absolute and relative power in various frequency bands, spectral entropy, spectral edge frequency, median frequency [133,159–161].

##### Bispectral analysis

In the case of the FFT analysis, the phase spectrum yields information about the phase of components in relation to the start of the computation window. The bispectrum measures the correlation of phase between different frequency components [129,162]. Additionally, bispectral analysis has other features like the suppression of Gaussian noise, thus enhancing the signal to noise ratio. Finally, the bispectral analysis is susceptible to non-linear relationships in the signal, which makes it very useful for processing the EEG signal.

### 4.3. Commercial DGA monitors

**Figure 4.**
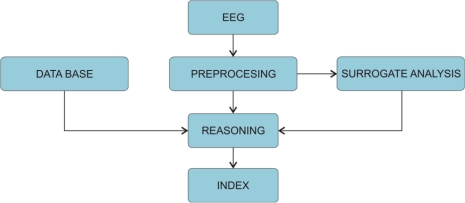
The conceptual diagram of a DGA monitor.

The first commercial EEG-mono-parameter was the Bispectral-Index or BIS, which was first introduced in 1992 and after 1999 others followed (e.g., Narcotrend, AEP-Monitor/2, PSA, CSM ...). These monitoring systems analyse the potential fluctuations measured from the patient’s forehead. After the signals are digitalised on the AD converters, the pre-processing unit determines which part of the signal is going to be analysed. Next, the artefact algorithm is applied to remove possible artefacts stemming from eye movement, swallowing or heart activity. The quality of the artefact removal algorithm is therefore crucial for the reliability of the DGA monitor. On the other hand, these artefacts are sometimes used as surrogate parameters, e.g., detection of swallowing determines the steady state of GA. Furthermore, the electromyogram (EMG) can also be used as surrogate parameter since the movements of the facial muscles are clearly visible in the frequency spectrum above 30 Hz. Consequently, the increase of the EMG surrogate parameter is interpreted as a decrease of the anaesthetic’s effect and vice versa. Next, algorithms of commercial monitors were trained and tested against the signals from the databases. The measured signal is then compared against the samples from the database and classified. Consequently, the index is as good as the database against, which it was trained. Finally, the calculated index values are post-processed to minimize fluctuations on the output. This is done by averaging over past index values, which stabilises the index, but adds delay into the index. Therefore, all commercial indices are: (a) constructed abstract quantities that are not directly linked to any physiological parameters and (b) have an inherent time delay (Figure 4). In the next sections, we will present some of the depth of anaesthesia monitors, which are in use in clinical practice and have endured some amount of clinical testing.

#### 4.3.1. BIS monitor

The BIS index was first introduced in 1992 by Aspect Medical Systems. The main component of the BIS monitor is the bispectral analysis, which evaluates the phase relations from a single channel EEG signal measured from the patient’s forehead. The BIS index is a dimensionless number from 0 to 100. The BIS algorithm is not publicly available; nevertheless, Rampil [131] disclosed some parts of the algorithm in 1998. Since the BIS algorithm is constantly updated, the BIS version must be checked, when evaluating its performance.

**Figure 5.**
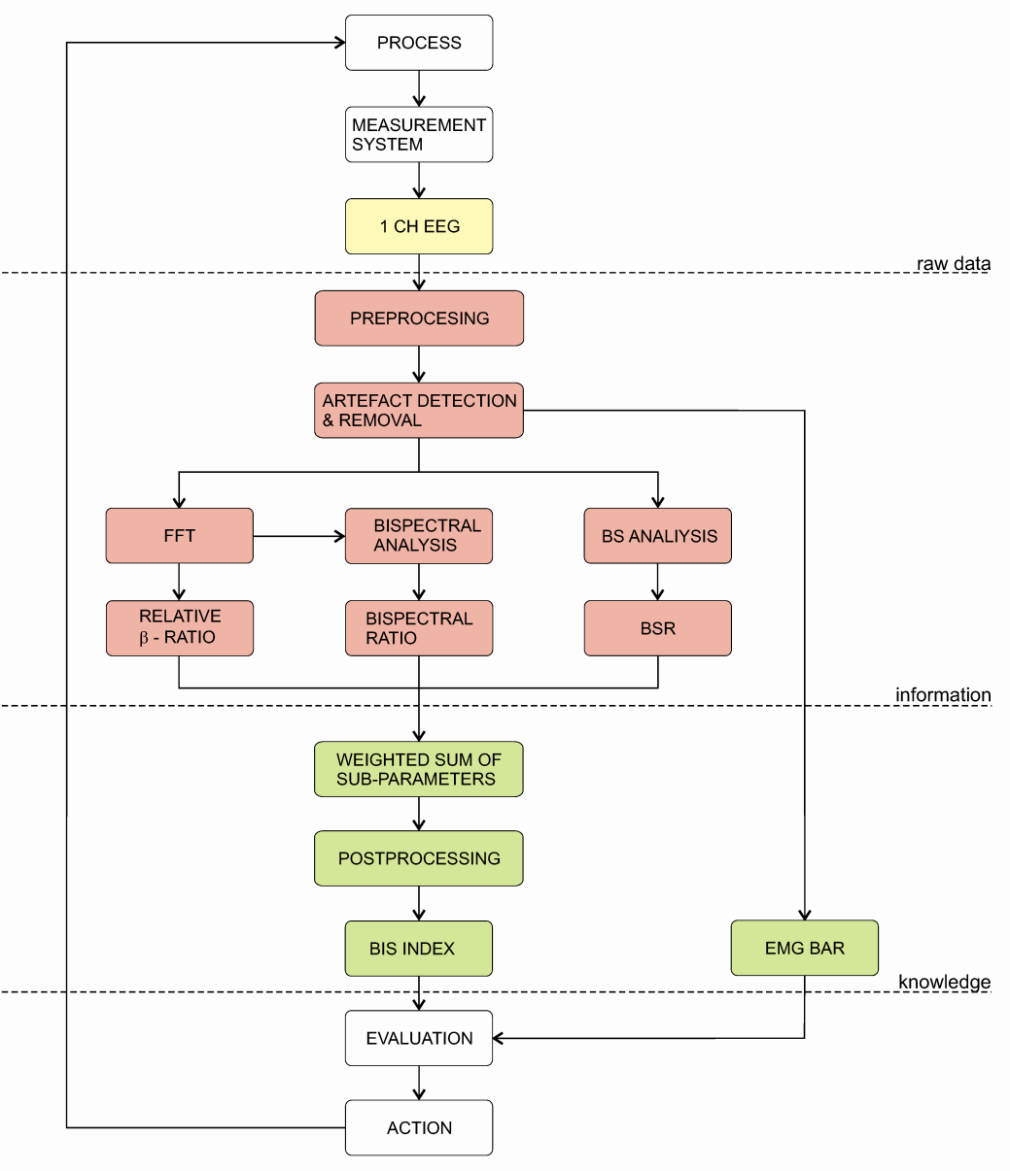
The block diagram of the BIS algorithm. Abbreviations: BSR (burst suppression ratio), electromyogram activity (EMG), FFT (fast Fourier transform).

The basic block diagram of the BIS algorithm is depicted on Figure 5. Firstly, the EEG signal is digitised and pre-processed. At this stage various algorithms for artefact detection and removal are applied. The most prominent sources of artefacts are: ECG, eye movement/blinking and power grid interferences. Additionally, BIS includes two types of BS detection. Firstly, the burst-suppression ratio (BSR) detects the portion of the isoelectric EEG in the last 60s, secondly, the QUAZI-Suppression detects BS patterns during power grid interferences, which can influence the BSR algorithm. Next, the pre-processed data is used to calculate the β-ratio parameter. This parameter is calculated as a ratio between empirically determined frequency bands (30–47 Hz and 11–20 Hz). Simultaneously, the second parameter, synch-fast-slow, is calculated from bispectral analysis. It is defined as a ratio between the sum of all spectral peaks between 0.5–47 Hz and the sum of all spectral peaks on the interval 40–47 Hz. Finally, all parameters are then fed to the weighting algorithm, which produces the BIS index [131–133,162].

#### 4.3.2. Narcotrend monitor

The Narcotrend monitor is produced by MonitorTechnik and was introduced in year 2000. The Narcotrend monitor classifies the state of anaesthesia into five stages, according to the work of Loomis *et al.* [138]. The stages (A–F) were further subdivided into three sub-stages by the work of Kugler [139]. Therefore, the Narcotrend Monitor can automatically distinguish between 14 and 15 stages (depending on the software version). Additionally, in analogy with BIS, the newer versions of the monitor also display the index value (0–100).

The development of the algorithm was based on a huge database of measurements, which were visually classified into five stages. Next, a set of parameters from time domain and frequency domain analyses were determined, e.g., several spectral parameters, spectral entropy and autoregressive parameters. These parameters were further statistically analysed to detect the parameters, which were the most suitable to discriminate between stages. Finally, visual inspection, classification and extraction of the parameters, were the foundations for the development of the classification function, which automatically classifies the EEG into the proposed stages.

**Figure 6.**
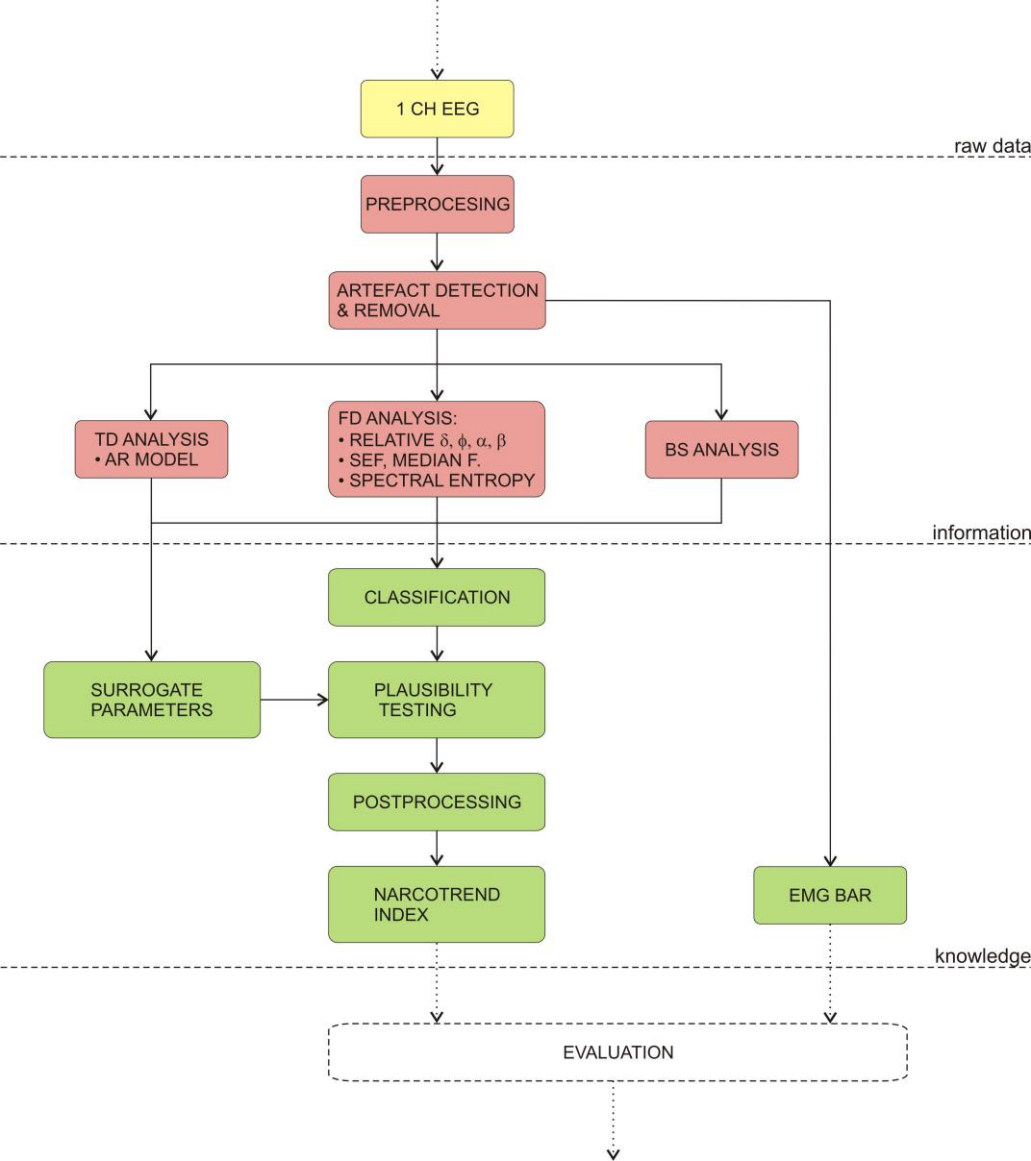
The block diagram of the Narcotrend algorithm. Abbreviations: BS (burst suppression), TD (time domain), FD (frequency domain), electromyogram activity (EMG).

Figure 6 shows the algorithm for calculating the Narcotrend index. Firstly, the EEG is recorded from three electrodes which are placed on the forehead. Secondly, the digitised signal is subjected to extensive artefact detection and removal algorithms. Then, it is analysed in the frequency domain on the frequency interval between 0.5 and 47 Hz. Next, the calculated parameters are passed to the classification function. Meanwhile, the monitor calculates the surrogate parameters, e.g., EMG, which are used in plausibility testing of the calculated index [134–136].

#### 4.3.3. AEP-Monitor/2

Many studies were performed, where the AEP were used to infer the level of GA. However, the first commercial monitor based on AEP was introduced by Danmeter in 2001. After further research, the same company introduced the new version of the monitor AEP-Monitor/2, which is not only based on AEP, but includes also spectral EEG parameters. The monitor uses autoregressive models with exogenous input (ARX) to detect the AEP. The ARX were used because the method enables fast responses of the monitor.

Like other successful DGA monitors the new version of the index calculates also the AEP-ARX-Index (or AAI—A-line autoregressive index), which is a dimensionless number. Unlike other monitors, AAI can be displayed on two scales: either from 0 to 100, or from 0 to 60; the second scale is recommended and the optimal anaesthesia is achieved if the index values are between 15 and 25.

**Figure 7.**
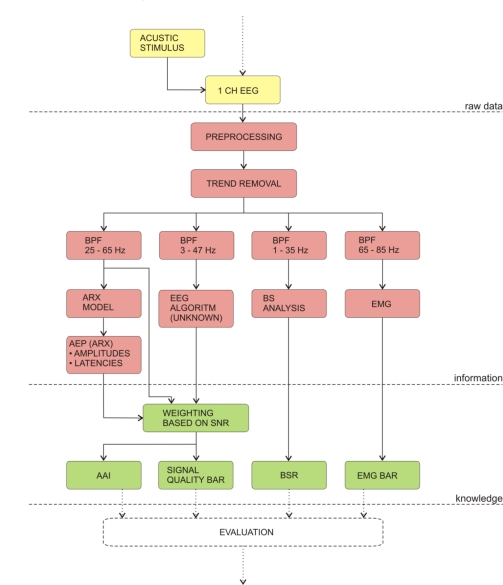
Block diagram of the algorithm of the AEP-monitor/2. Abbreviations: ARX (autoregressive models with exogenous input), BS (burst suppression), BSR (burst suppression ratio), BPF (band pass filter), EMG (electromyogram activity), AAI (AEP-ARXI), SNR (signal–to–noise ratio).

The algorithm of the AAI index is shown on Figure 7. First, the monitor records EEG, while the short bilateral clicks (2 ms) are emitted through headphones. Second, the monitor digitizes the signal and detects and removes artefacts. Next, the signal is put through several band-pass filters (BPF) to detect various features in different frequency bands. The AEP are detected in the frequency range 25–65 Hz, with the use of ARX. The reconstructed amplitudes and latencies are evaluated after the ARX analysis. Meanwhile, the analysis of the EEG in frequency band between 3–47 Hz is performed by an undisclosed algorithm. Because the EMG is propagated in the same frequency band, the AAI can be influenced by the EMG artefacts. Next, the decision how the AAI is constructed from AEP and EEG analyses is made by the weighting function, which is based on the signal–to–noise ratio. Next, the AAI is displayed on the front panel of the instrument. Along with the AAI, additional EEG parameters are displayed: the signal quality bar shows the user how the AAI is constructed from its components. For additional information, the burst suppression ratio and EMG bars are also displayed alongside the AAI. All of the above parameters must be monitored simultaneously in order to ensure optimal sedation of the patient during GA [140–143,148].

#### 4.3.4. PSA 4000 Monitor

**Figure 8.**
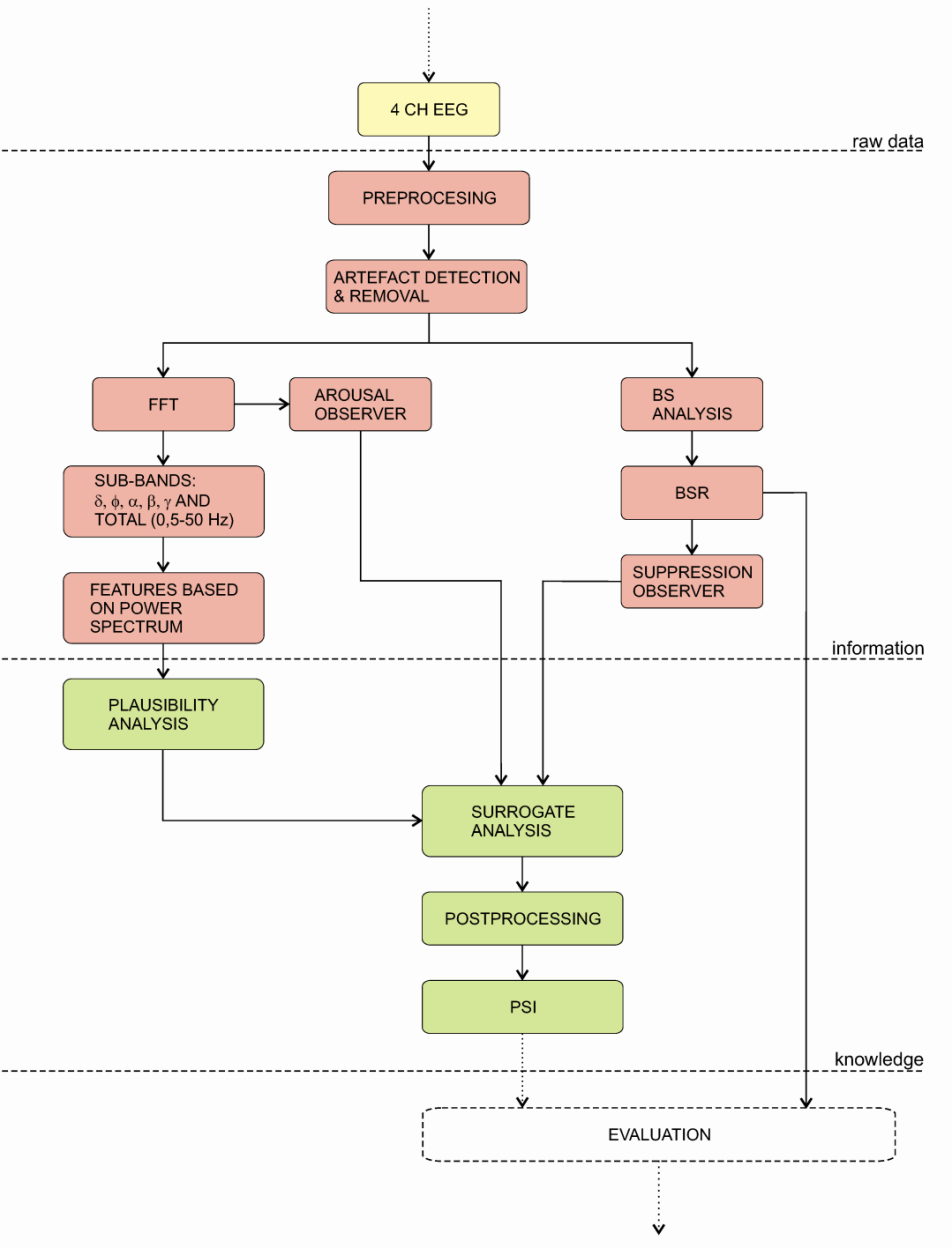
The block diagram of the algorithm of the PSA 4,000 monitor. Abbreviations: BSR (burst suppression ratio), FFT (fast Fourier transform), PSI (patient state index).

The Patient State Analyser 4000 (PSA) was developed by the Physiometrix Company and launched in 2001 in the USA. Unlike other indices the PSA monitor calculates the value of the index from four EEG channels (see Figure 8). The acquired signals are pre-processed and an artefact removal algorithm is applied. Next, the frequency domain analysis is applied to calculate several features of specific EEG frequency bands: δ, θ, α, β, γ and of total EEG frequency band (0–50 Hz).

The PSI uses a set of features that best describe the variances, which are related to the EEG. These features were derived from the analyses of databases obtained in previous studies. The databases contain a huge number of EEG measurements and were used to: (a) develop an artefact detection algorithm, (b) determine features for description of anaesthesia states and conditions and (c) calibrate the PSI index. Next, the set of features found to account for most statistical variance related to hypnotic state (selected from database recordings) were derived for input to the discriminant algorithm. These features include measures such as: (a) absolute power gradient between frontpolar and vertex regions in γ, (b) absolute power changes between midline frontal and central regions in β and between midline frontal and parietal region in α, (c) total spectral power in the frontpolar region, (d) mean frequency of the total spectrum in midline frontal region, (e) absolute power in the δ band at the vertex and (f) posterior relative power in slow δ.

All features are included in a plausibility analysis to calculate the Patient State Index (PSI), which is a dimensionless number from 100 (awake) to 0 (isoelectricity). Additionally, surrogate analysis is performed by calculating two parameters: BS and arousal detection. This means that both parameters modulate PSI index in the case when the signal quality is under question. Finally, before the index is displayed, it is post-processed with an averaging algorithm, to provide a more stable output [144,145].

#### 4.3.5. IoC Monitor

**Figure 9.**
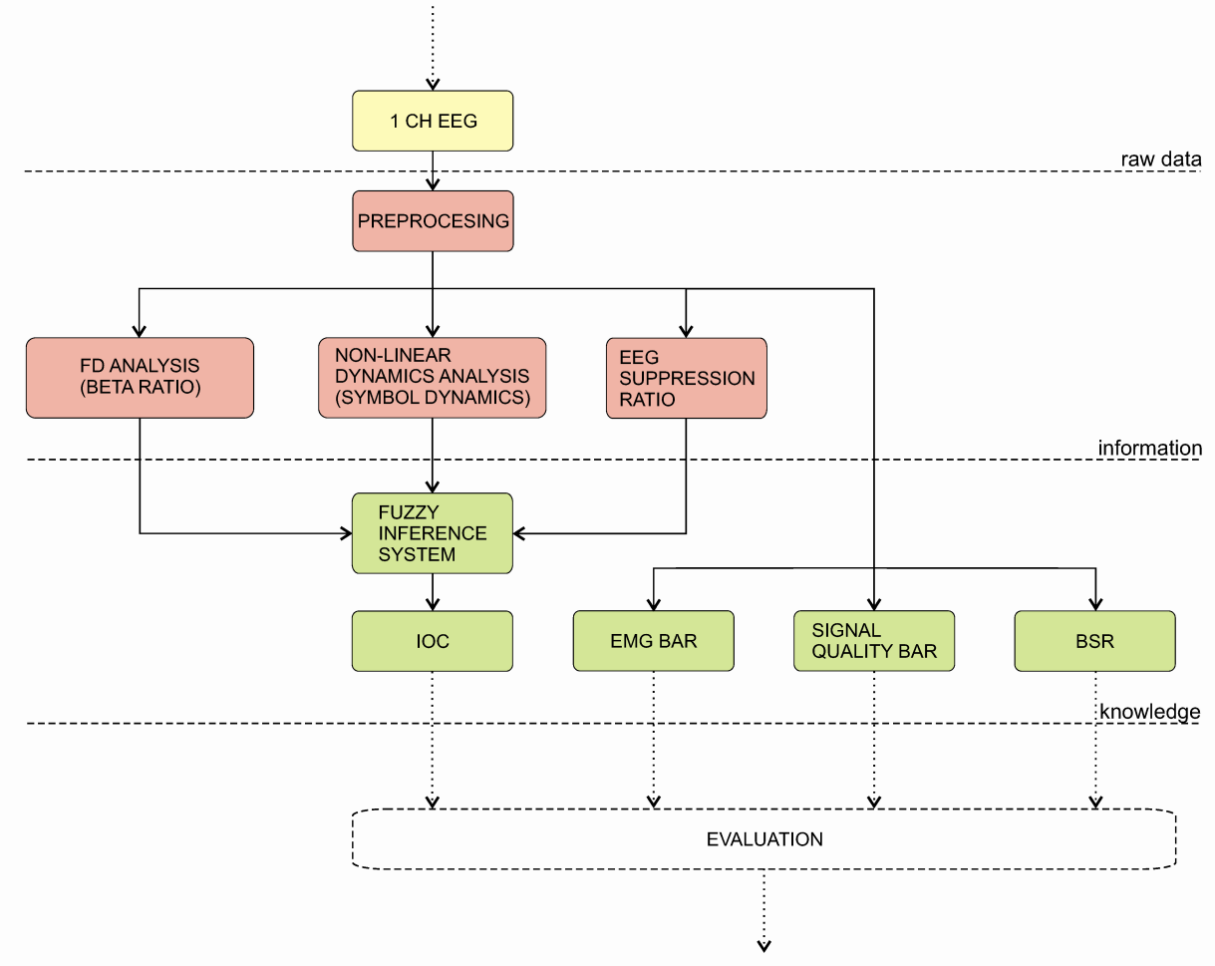
The block diagram of the IoC’s algorithm. Abbreviations: BSR (burst suppression ratio), FD (frequency domain), electromyography activity (EMG).

The Index of Consciousness (IoC) monitor was introduced by the Morpheus Medical Company. The IoC records the electroencephalogram (EEG) with thee surface electrodes attached to the patient’s forehead. The main parameter of the IoC is the symbolic dynamics method, which divides EEG into a finite number of partitions and assigns a symbol to each partition. The alternation of these symbols determines the dynamics of the EEG. The symbolic dynamics detects the complex non-linear properties of the EEG that can be correlated to the depth of anaesthesia [146]. The IoC monitor incorporates additional parameters: β-ratio and EEG BSR. All these parameters are then combined through a set of fuzzy logic rules into a single index. Besides IoC, the monitor displays EMG bar signal quality bar and BSR. The algorithm is depicted on Figure 9. [146,147].

#### 4.3.6. Cerebral State Monitor

**Figure 10.**
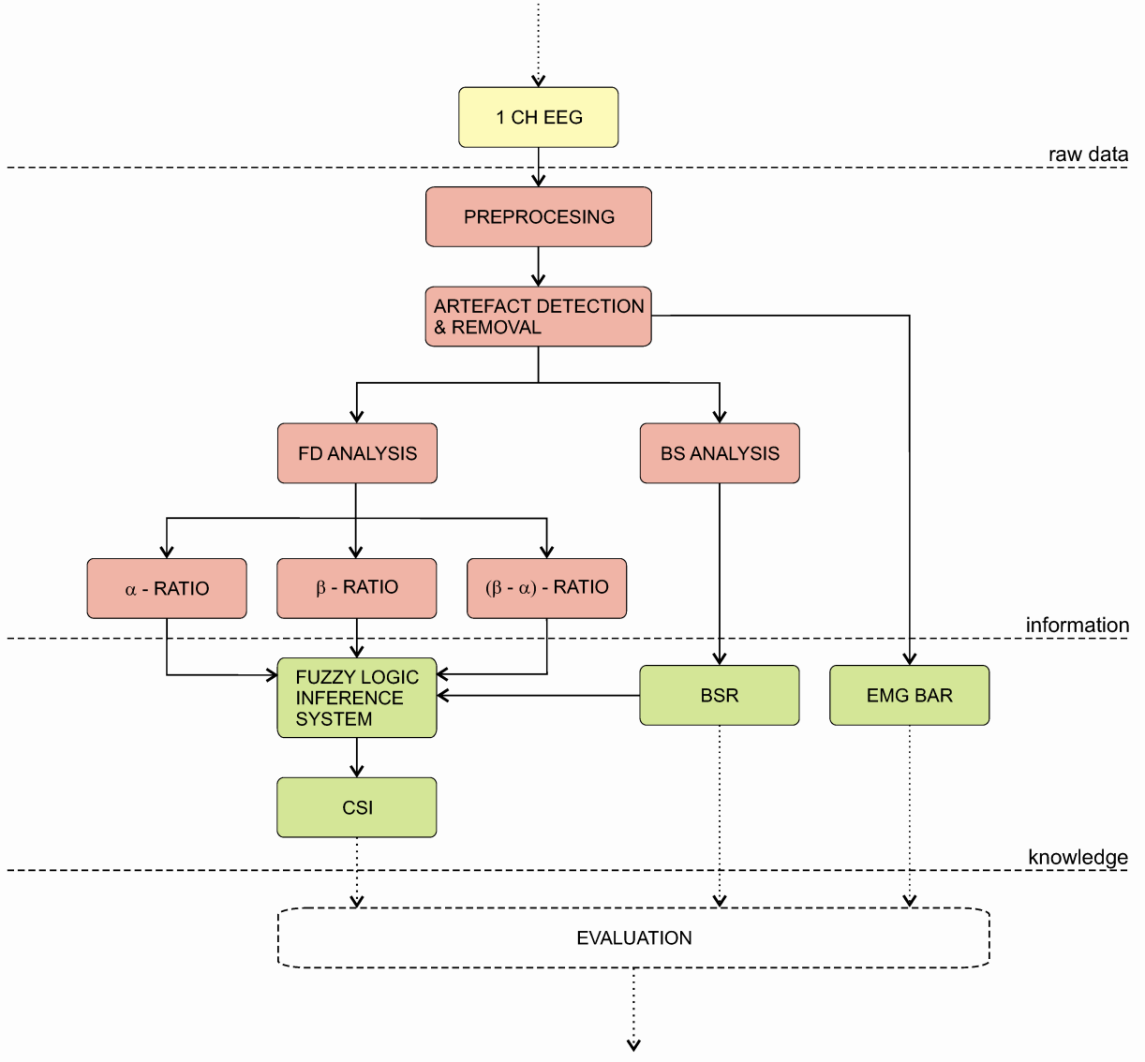
Block diagram of the algorithm of the CS Monitor Abbreviations: BS (burst suppression), BSR (burst suppression ratio), FD (frequency domain), electromyogram activity (EMG), CSI (cerebral state index).

The Cerebral State Monitor (CSM) was introduced by the Danmeter Company in 2004. The monitor’s algorithm is build upon the EEG-algorithm of its predecessor AEP-Monitor/2 and uses the same electrodes. The CSM is a portable, wireless monitor that uses fuzzy logic to calculate the cerebral state index (CSI). The algorithm is depicted on Figure 10. Firstly, one channel EEG signal is pre-processed and then processed by an artefact detection and removal algorithm. The pre-processed signal is then used to calculate three parameters in frequency domain: (a) α–ratio, (b) β–ratio and (c) α–β-ratio. Additionally, the BS analysis is applied to calculate the BSR. Afterwards, all parameters are fed to a fuzzy logic inference system to calculate the cerebral state index (CSI). Finally the CSM displays the CSI, BSR and EMG values [148].

#### 4.3.7. Entropy-Module

The Entropy Module was introduced in 2003 by the Datex-Ohmeda Company. The product is based on many studies [150,151,161], which show that entropy could be used to characterise the EEG and the depth of GA. The main idea is that increasing depth of anaesthesia causes increase in regularity of the EEG, which can then be inferred by the entropy and used to estimate the depth of anaesthesia.

**Figure 11.**
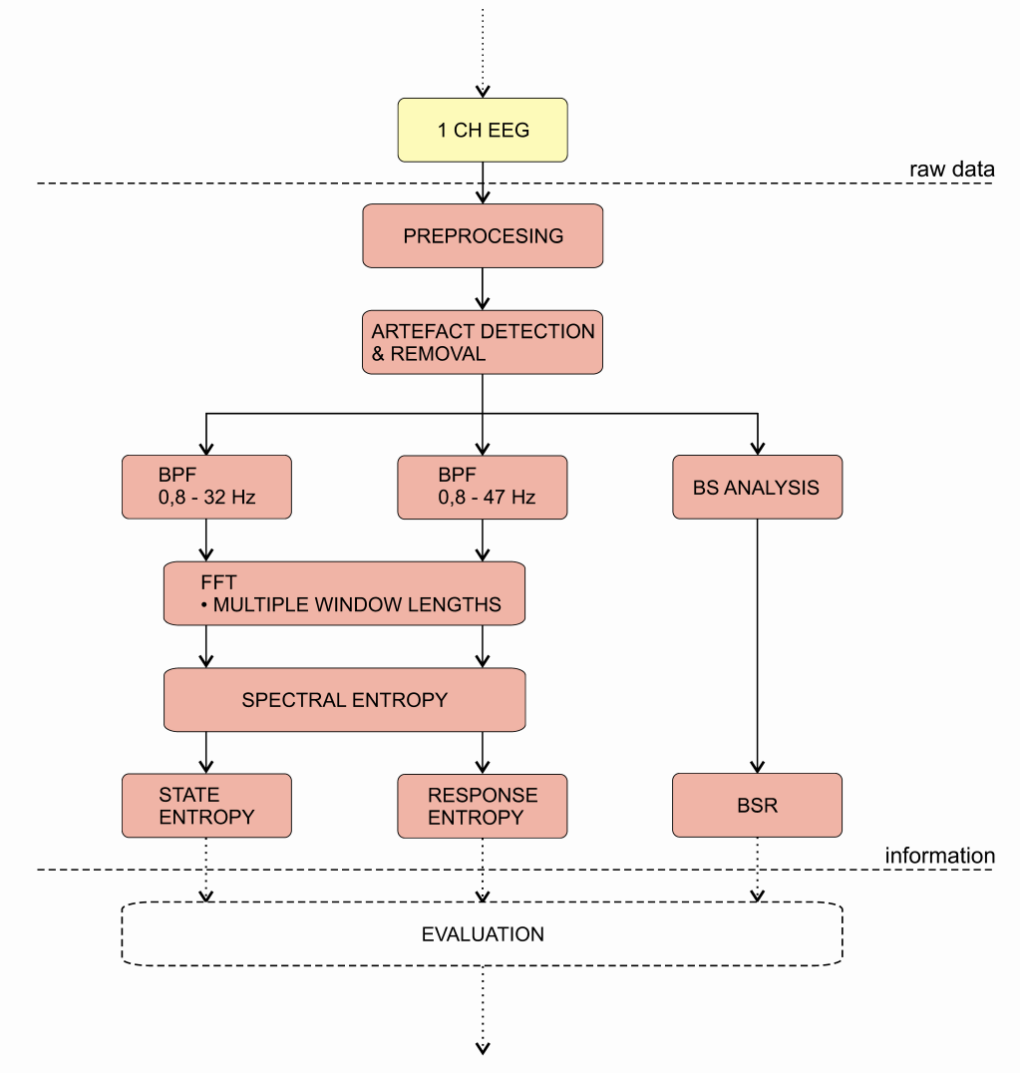
Block diagram of the Entropy module algorithm. Abbreviations: BS (burst suppression), BPF (band pass filter), BSR (burst suppression ratio), FFT (fast Fourier transform).

A block diagram of the monitor’s algorithm is presented on Figure 11. The first step of index calculation is the digitalisation of a single channel EEG. Then, the monitor applies pre-processing algorithms. Next, the digitised signal is put through the artefact detection and removal algorithm. After that, the signal is divided into two frequency bands (0.8–32 Hz and 0.8–47 Hz).

The FFT of the signals is performed with various window lengths. This technique is similar to the multi-scale analysis, e.g., wavelet analysis, where different window lengths ensure optimal resolution for each frequency band. The spectral entropy is then calculated from the power spectrum of the particular epoch of the signal within a particular frequency band. According to the aforementioned frequency bands, two features are calculated: state entropy (SE) and response entropy (RE). Both are dimensionless numbers between 91-0 and 100-0, respectively. Additionally, the monitor performs the BS analysis and displays the BSR. The main difference from other DGA monitors is that this monitor outputs two index values. While the SE includes information only from EEG, the RE also includes the EMG activity and can be therefore used as a surrogate parameter. Consequently, the difference between indices, which is larger than 10 indicates increased muscle activity. In conclusion, the monitor does not perform any fusion of the calculated features into a single index like in the previous cases (e.g., BIS). Therefore, the interpretation of the results is left entirely to the anaesthetist [79,149,150].

## 5. Comparison of the Technical Properties among DGA Monitors

In this section we discuss the key qualities that are shared among the DGA monitors, as well as the differences that may be important for the performance of a DGA monitor in clinical practice. These qualities are summarised in Table 2.

### 5.1. The use of a priori knowledge in development of the decision making algorithm

The iterative process in development of the index algorithm improves its quality and reliability. In this process the algorithm should be refined against a high quality database with expert interpretation. In this regard several publications exist about the development of the BIS, Narcotrend and PSA monitor algorithms [131,134,144]. BIS was developed in an iterative process, where the discriminating parameters were tested for performance against a database of EEG measurements related to different anaesthesia states [131]. The Narcotrend algorithm was developed in a similar fashion, where the database was visually inspected and classified into stages. The classified data then served to extract the time and frequency domain features to be used in classification function [134,137]. The development of the PSI algorithm was based on three databases of EEG records, which were used for development of artefact detection and removal algorithms, classification algorithms and finally a third database was used for calibrating the index [144]. On the other hand little is known about the inclusion of prior knowledge in the development of other indices (AAI, SE, CSI and IoC). This does not mean that the monitors were not tested and calibrated against real EEG signals, however, the size of the database and the development process are an important property of the DGA monitor and can reflect the discriminating power of the index.

### 5.2. Features for calculating the DGA indices

After the pre-processing stage of the data analysis all indices calculate features, which are used for index calculation. The number and type of features vary from monitor to monitor, although, most of the features are established empirically and do not reflect any underlying determinism. Exceptions are BIS, Narcotrend and AEP-Monitor/2 (AAI). The BIS performs a bispectral analysis of the EEG signal and analyses the phase relationships of the EEG components. Here, a weak assumption of the model can be made, that the phase relationships are connected to the number of different oscillatory generators in the brain [131]. The Narcotrend uses autoregressive (AR) modelling to infer different features [134,148]. Also, the AEP-Monitor/2 uses AR models to infer AEP from the EEG [140]. However, AR models are only used for modelling statistical properties of the signal; therefore, it is not possible to infer the underlying dynamics of the signal. At the same time, Narcotrend uses a set of empirical features based on spectral power: spectral edge frequency and β-ratio.

A similar approach was used for the PSA, where a set of spectral features were established from database EEG records [144]. The same approach is also used by the CSM, which is based on α and β power ratios [148]. An alternative approach is used by the Entropy Module (SE and RE) and the IoC Monitor. In the first case the spectral entropy calculation is combined with multi-resolution analysis, which yields an optimal time-frequency resolution. The entropy can be regarded as an estimator of signal complexity and is assumed to be related with the DGA [150,151]. Moreover, it can detect non-linear features in the signal. A similar principle is used by the IoC monitor, which analyses the symbol dynamics of the EEG signal. Finally, while the indices mostly differ in the number and type of features used, the BSR is calculated by all monitors and is included either in the index calculation algorithm, or is displayed as additional information for the anaesthetist [18,148].

### 5.3. Algorithms for calculating the DGA indexes

After the feature extraction is completed, each monitor uses a different algorithm to calculate or classify the DGA. BIS calculates its value by a simple weighted sum of features [131]. The weighing function is most probably modulated by the state of anaesthesia, since a linear dependence on BSR in deep anaesthesia has been demonstrated in [132]. Narcotrend monitor uses a classification function, which derives the state of anaesthesia from the extracted features. Additionally, the Narcotrend monitor calculates additional surrogate parameters, which are sensitive to atypical patterns of general anaesthesia, e.g., K complexes [134]. These parameters are then used in a plausibility analysis before the index is displayed. This is the probable reason for Narcotrend’s better performance in comparison with BIS regarding the susceptibility to increased EMG activity [152]. Similarly, the AEP-Monitor/2 (AAI) also has a weighting function to calculate the index from the ARX model. The weighting function is modulated by the signal quality and the presence of artefacts. Although, the modulation function implies that the influence of the artefacts on the final index is accounted for, the values of AAI and BIS have decreased significantly in a study where muscle relaxants were administered to the ICU patients [153,155]. The PSA first conducts a plausibility analysis to classify the state of anaesthesia from the values of spectral features. Additionally, it performs surrogate analysis to test against artefacts and BS. A specific approach has been taken by the Entropy Module, where the monitor uses no special inference algorithm but rather relies on two normalised spectral entropy values: state entropy (SE) and response entropy (RE). The authors argue that artefacts like EMG should be treated as a signal and are therefore included in the RE number [79]. The anaesthetist should look for a difference between the SE and the RE, where a difference larger than 10 indicates the presence of significant EMG interference in the EEG signal. The IoC Monitor and CSM both use a fuzzy logic set of rules to produce an index. Although the CSM includes the artefact control function, the features are calculated on the frequency bands which could include EMG activity. Although, a comparative study showed that EMG had no effect on CSM or BIS index in children, an open question remains how much influence the EMG has on the CSM in adults [156]. Additionally, a case report indicates a strong correlation between EMG and CSM in an ICU patient [154]. Finally, the IoC Monitor, which also calculates the index via a fuzzy inference system, excludes the EMG variable from the algorithm. Alternatively it displays the EMG value separately and it is up to the anaesthetist to decide how to react to the increased EMG values. The authors stress the fact that a sudden change in the IoC, accompanied by a rise of EMG, is probably the effect of increased muscle activity [147].

### 5.4. The response time of DGA monitors

One of the most important parameters of a DGA monitor is its response time (*i.e*., time delay) to a rapid change in the level of sedation. Unfortunately, not many studies have been performed where this parameter was tested. Zanner and co-workers used recorded EEG data to test the responsiveness of the DGA indices [157]. The data were selected to represent an awake state, a general anaesthesia and a suppression of cortical activity. Then the authors measured the response time of BIS and Narcotrend monitors to these EEG signals. The study reported time delays between 26 ± 7 seconds and 106 ± 23 seconds for the transition between EEG suppression and the awake state for BIS, Narcotrend and CSM (see Table 2 for details). Additionally, the study showed that the time delays were not constant and were dependent upon the depth of GA. Also, the response times were different for the increasing and decreasing states of GA. Next, a similar study was performed, where the authors used simulated EEG signals on the same monitors [158]. The time delays fluctuated between 14 seconds and 155 seconds, also, the rise and fall times did not match for the same DGA index. Therefore, both studies raise concerns about the timely detection of rapid EEG changes concomitant with the transition from general anaesthesia to awareness. Additionally, these findings limit the use of DGA monitors for pharmacodynamic modelling. Similar studies for other DGA indices have not been published to date.

### 5.5. Advantages and disadvantages of different DGA monitors

Despite the abundance of studies on commercial DGA monitors, it is still difficult to compare their performance because there is no consensus on the validation methodology [19] and the majority of studies have been performed on the minority of DGA monitors (*i.e.*, BIS, Narcotrend and Entropy module). Despite the aforementioned problems, the fact that the BIS monitor has been present for almost two decades and has been evaluated by many studies, makes it a *de facto* standard against which all other DGA monitors are compared. The BIS and Entropy monitor are the only DGA monitors that have been routinely used in child anaesthesia [163–168]. However, this does not necessarily mean that the BIS monitor is superior to other DGA monitors in all other aspects of anaesthesia monitoring.

Some conclusions can be made about the performance of individual DGA monitors by analyses of the inferred characteristics of their DGA algorithms (only conceptual descriptions of DGA algorithms were published, no DGA algorithm has been published with full technical specifications so far). The influence of various interferences on DGA index can be studied by checking for the presence of surrogate analysis in the respective algorithm. It can be expected that BIS would have more problems with EMG interference than Narcotrend, which was shown in [169]. This is most likely due to the fact that Narcotrend modulates the index value with artefact surrogate analysis. Susceptibility to EMG interference is expected in monitors that have a DGA algorithm similar to the PSA monitor. The performance of artifact removal algorithms was tested in [170], where it was shown that Narcotrend’s artifact removal algorithm was more likely to exclude the data from further analysis, due to the presence of artifacts. Unfortunately, no such claims could be made for other monitors, due to lack of similar studies. The complexity of the algorithm is an important property, which also influences the time delay or the response time of a monitor to an abrupt change in sedation. Here BIS, IoC and Entropy Module DGA monitors are advantageous, since their algorithms are simple, that is, they include few FD or TD features. This fact was shown in recent articles [171,172], where BIS outperformed the Narcotrend, PSI and CSM monitor. Here, despite the fact that CSM has a simpler algorithm in comparison to Narcotrend, it was the slowest to respond to a change in sedation. Burst suppression is an important indicator of the level of deep sedation. The BSR is therefore included in every DGA monitor. The way it is incorporated into the algorithm might have the influence on the way monitors react different or extreme conditions like oversedation. The monitors like BIS, Narcotrend, IoC, CSM might have an advantage, since the BSR analysis is directly included in the index calculation, while on the other hand, monitors like AEP-Monitor/2, PSA and EntropyModule use BSR analysis in surrogate analysis to modulate the value of the index or in some cases display the value of BSR independently. This fact was reflected in the study [173], where BIS outperformed the PSI (PSA) in the prediction of oversedation.

Recently, two studies compared the monitoring of GA with the BIS and the CSM (marketed as a low cost DGA monitor) during propofol/remifentanil [174] and propofol/fentanil anaesthesia [175]. In the first study, the CSM was a satisfactory alternative to the BIS for monitoring hypnotic effect in 87% of patients; in 13% of the patients, the CSM displayed values indicating an awake state despite clinical sleep, all correctly identified with the the BIS [174]. In the second study, propofol anaesthesia guided with the CSM resulted in 20% higher propofol doses compared with the BIS; there were no clinically relevant differences in recovery times [175].

The DGA monitoring technology is promising and used widely, nevertheless to date no health authority has so far recommended that such monitors should be compulsory during general anaesthesia, but should be considered only on an individual basis [176].

## 6. Towards a Better DGA Monitor

There is a growing interest in EEG-based DGA monitors, which is illustrated by the increasing number of new DGA monitors that are used in clinical practice. The pace of development of future DGA monitors will depend on two types of changes: changes in the validation process and the introduction of new concepts in the design of mathematical algorithms that interpret EEG signals during GA. The validation process of EEG or AEP derived indices of aesthetic depth would be accelerated if a consensus could be reached on a validation protocol [19]. Most likely, such a validation process would have to be developed and coordinated by an international professional body. Several algorithms were developed for the calculation of EEG or AEP derived metrics but most have not been published fully or not at all. The practice of using open-source algorithms for development of future DGA monitors would accelerate the improvement of existing DGA monitors and hasten the arrival of the next generation [20]. The key challenge that faces the developers of DGA monitors is the design of algorithms that measure the transition from consciousness to shallow and then to deep anaesthesia independently of the type and concentration of a general aesthetic needed for these transitions to occur. Current DGA monitors do not measure the state of consciousness directly, but can still assess quite well the transition point when loss of consciousness occurs for most general anaesthetics with the exception of ketamine, nitrous oxide or xenon [19,20]. For the same reason no currently available DGA monitor distinguishes among the waking state, anaesthetic- induced unconsciousness, REM, and non-REM sleep. This deficiency has important practical consequences—*i.e.*, it is not always possible to detect a sleeping patient before surgical stimulation causes arousal and awareness [19]. A potentially promising way forward, in the development of algorithms for monitoring the DGA, would be the correlation of information integration in the brain with different stages of unconsciousness [20]. As discussed previously, the loss of consciousness during GA seems to be associated with a breakdown of cortical connectivity with concomitant, quantitative and qualitative changes in the flow of information [1,21].

To maintain an appropriate depth of GA, anaesthesiologists routinely increase the delivered anaesthetic concentration in anticipation of increased surgical stimulation. Röpcke and co-workers systematically studied the EEG concentration-response relationship of desflurane and concluded that surgical stimulation shifted the desflurane concentration—electroencephalographic effect curves for the BIS index toward higher desflurane concentrations [177]. DGA monitors discussed in this review are essentially monitoring only the hypnotic component of GA and do not evaluate the patient’s stress level during surgery (*i.e.*, the nociceptive component of GA). For example, during GA maintained with desflurane or propofol, a surgical incision (a painful stimulus) has modest effects on the EEG patterns; these effects are not strongly modified by the depth of anaesthesia as estimated by the BIS [178]. The monitoring of stress response during surgery is important, because prolonged surgical stress can lead to increased morbidity and delayed postoperative recovery [179–181]. Surgical stress reflects the balance between nociception—the neural processes of encoding and processing painful stimuli and anti-nociception—a reduction in pain sensitivity produced within neurons for example due to the application of drugs that relieve pain. Traditionally, the changes in heart rate and arterial pressure were used as signs of increased nociception during GA but their specificity and sensitivity is not very high [182,183]. Changes in skin conductance [184] or the number of skin conductance fluctuations per second [185] are reported to be more useful for estimating surgical stress then heart rate or arterial pressure. Recently, several multivariable approaches for measuring the nociception—anti-nocipection balance were developed: the surgical stress index (SSI), the response index of nociception (RN) and the noxious stimulation response index (NSRI). The NSRI is computed from hypnotic and opioid effect-site concentrations using a hierarchical interaction model; it is reported to better predict the analgesic component of anaesthesia than the BIS or AAI index or predicted propofol or remifentanil concentrations [186]. The RN algorithm combines information on the patient’s reactions and clinical signs of nociception, remifentanil levels and estimation of noxious intensity of surgical incision into a clinical score from 0 to 100 [187]. The SSI is based on a sum of ther normalized pulse beat interval and the pulse wave amplitude time series of photopletismography. SSI appeared to be a better measure of the nociception—anti-nocipection balance than state and response entropy, heart rate or pulse wave amplitude [188]. Prospective validation studies have yet to be done for all three indices.

## 7. Conclusions

General anaesthetics have been used in medicine for more than a century without a clear understanding of the underlying physiological mechanisms that regulate the transition from a wake state to an anaesthetic-induced unconsciousness. Recent advances in the field of neurobiology and the introduction of EEG based DGA monitors have made important contributions towards our understanding of fundamental changes in brain activity brought about by general anaesthetics. Current DGA monitors directly measure the concentration-dependent affect of a limited number of general anaesthetics on the brain and indirectly the state of consciousness during GA; also they do not evaluate the patient’s stress level during surgery (*i.e.*, the nociceptive component of GA). The development of a true DGA monitor, which directly measures the state of consciousness and also the nociceptive component of GA, would enable, not only a safer and more cost-effective anaesthetic procedure, but would also provide us with a key to a deeper understanding of the essence of human being, the one that defines us with respect to other living beings, and would give the centuries old philosophical statement *Je pense donc je suis—I think, therefore I am* a profoundly new meaning.

## Figures and Tables

**Table 1.** Basic characteristics of EEG wave bands.

**EEG bands**	**Frequency (Hz)**	**Amplitude (mV)**	**Generator of brain waves**
**α (alfa)**	8–13	20–60	thalamus
**β (beta)**	13–30	2–20	cortex
**γ (gama)**	30–70	3–5	thalamus
**δ (delta)**	0,5–4	20–200	talamus
**θ (theta)**	4–7	20–100	hippocampus and neocortex

**Table 2.** Comparison of DGA monitors.

	**BIS**	**Narcotrend**	**PSA 4000**	**AEP-Monitor/2**	**Entropy Module**	**CSM**	**IoC**
Database included in the development of algorithm or for inferrence	Yes	Yes	Yes	No; index based on previous studies of the algorithm	No; index based on previous studies of the algorithm	No; index based on previous studies of the algorithm	No; index based on previous studies of the algorithm
Features or methods included in algorithm	Bispectral analysis, beta-ratio	SEF, median fr., spectral entropy, relative δ,θ,α,β, AR model	Several frequency domain features extracted from power spectrum	AEP, ARX model	Multiscale analysis, entropy, spectral entropy	α, β, α-β power ratios	Symbol dynamics analysis
Surrogate analysis	No	Yes	Yes	Yes	No	No	No
Burst suppression analysis	Yes	Yes	Yes	Yes	Yes	Yes	Yes
Index calculation	Weighted sum of subparameters	Classification function with plausibility analysis	Plausibility analysis with surrogate testing against BSR and arousal parameters	Modulation of index based on SNR and EMG	Entropy, no inference algorithm	Fuzzy logic inference system	Fuzzy logic inference system
Estimated time delay (deep anaest. → awake, awake → deep anaest.)	63 s / 61 s	90 s / 26 s	Data not available	Data not available	Data not available	106 s / 55 s	Data not available
Susceptibility to EM interference	Moderate	Moderate	Data not available	Data not available	High	Moderate	No data available
Agreement with clinical signs of anaesthesia	Yes	Yes	More studies needed	Yes	Yes	Yes	More studies needed
Appropriate for ketamine or N_2_O anaesthesia	No	No	No	No	No	No	No data available
Papers on adult anaesthesia cited in Pubmed	878	53	5	19	109	21	2
Papers on child anaesthesia cited in Pubmed	122	8	2	5	3	4	0
Outcome improvement	Yes, but more studies needed	Yes, but more studies needed	Data not available	Data not available	Data not available	Data not available	Data not available
Cost effectiveness	For selected high risk patients	Data not available	Data not available	Data not available	Data not available	Data not available	Data not available
